# Genetic and Metabolite Diversity of Sardinian Populations of *Helichrysum italicum*

**DOI:** 10.1371/journal.pone.0079043

**Published:** 2013-11-18

**Authors:** Sara Melito, Angela Sias, Giacomo L. Petretto, Mario Chessa, Giorgio Pintore, Andrea Porceddu

**Affiliations:** 1 Dipartimento di Agraria, Università degli Studi di Sassari, Sassari, Italy; 2 Dipartimento di Chimica e Farmacia, Università degli Studi di Sassari, Sassari, Italy; 3 Centro Interdipartimentale per la Conservazione e Valorizzazione della Biodiversità Vegetale, Loc. Surigheddu, Sassari, Italy; George Washington University, United States of America

## Abstract

**Background:**

*Helichrysum italicum* (Asteraceae) is a small shrub endemic to the Mediterranean Basin, growing in fragmented and diverse habitats. The species has attracted attention due to its secondary metabolite content, but little effort has as yet been dedicated to assessing the genetic and metabolite diversity present in these populations. Here, we describe the diversity of 50 *H. italicum* populations collected from a range of habitats in Sardinia.

**Methods:**

*H. italicum* plants were AFLP fingerprinted and the composition of their leaf essential oil characterized by GC-MS. The relationships between the genetic structure of the populations, soil, habitat and climatic variables and the essential oil chemotypes present were evaluated using Bayesian clustering, contingency analyses and AMOVA.

**Key results:**

The Sardinian germplasm could be partitioned into two AFLP-based clades. Populations collected from the southwestern region constituted a homogeneous group which remained virtually intact even at high levels of *K*. The second, much larger clade was more diverse. A positive correlation between genetic diversity and elevation suggested the action of natural purifying selection. Four main classes of compounds were identified among the essential oils, namely monoterpenes, oxygenated monoterpenes, sesquiterpenes and oxygenated sesquiterpenes. Oxygenated monoterpene levels were significantly correlated with the AFLP-based clade structure, suggesting a correspondence between gene pool and chemical diversity.

**Conclusions:**

The results suggest an association between chemotype, genetic diversity and collection location which is relevant for the planning of future collections aimed at identifying valuable sources of essential oil.

## Introduction

The genus *Helichrysum* (Asteraceae, Gnaphalieae) includes over 500 species, distributed worldwide [1]. The species are highly diverse with respect to both phenotype and metabolite profile [2]–[4]. *H. italicum* (Roth) G. Don is a typical endemic Mediterranean species, able to colonize environments ranging in altitude from zero to 2,200 m a.s.l. [2], [5]. It has been sub-divided into ssp. *italicum* (distributed as isolated populations in Morocco, Cyprus, Corsica, some Aegean islands and Italy) [6], ssp. *michrophyllum* (Willd.) Nyman (present in the Balearic Islands, Corsica, Crete and Sardinia) [5] and ssp. *siculum* (Jord. & Fourr.) Galbany, L. Sáez & Benedı (endemic to Sicily) [7]. Both ssp. *italicum* and ssp. *michrophyllum* are found throughout Sardinia, from sandy beaches to holm oak forests at an altitude of 1,250 m a.s.l. Although certain morphological traits have been proposed to discriminate between the two subspecies, their phenotypic plasticity has caused problems in their taxonomic assignment. A number of molecular marker studies have attempted to address this taxonomic problem [5], [6], but little attention has as yet been paid to characterizing the relationship between intra-specific genetic diversity and either growing environment or metabolite profile.

The species complex has attracted attention on account of its secondary metabolite content, specifically flavonoids, sesquiterpene lactone and essential oils [3], [8]–[13]. *H. italicum* extracts have been shown to exhibit both antioxidant and anti-inflammatory activity [14], [15], and its antimicrobial activity (against both *Staphylococcus aureus* and *Candida albicans*) has been ascribed to the presence of terpenes and terpenoids [16]–[18].

The composition of essential oils is known to depend both on the collection site and on the developmental stage of the plant. The essential oil profiles derived from a set of Corsican and Tuscan ssp *italicum* accessions produced two distinct groups, with the profiles of the Corsican oils being dominated by neryl acetate, neryl propionate, nerol, acyclic ketones and β-diketones, while that of the Tuscan ones featured α-pinene and β-caryophillene. Meanwhile, ssp. *microphyllum* accessions collected in Sardinia and Corsica showed a similar composition, characterized by a high content of neryl acetate, nerol and neryl proponiate [19]. In spite of some sustained effort directed towards characterizing the metabolite profile of *H. italicum*, little emphasis has as yet been given to linking genetic with metabolite diversity [20], [21]. Such information would be useful in the context of formulating rational collection strategies.

Based on a combination of molecular marker and morphological evidence, it has been argued that Corsican and Sardinian populations of ssp. *italicum* and ssp. *microphyllum* share the same gene pool [5]. The high levels of morphological diversity observed have been seen as reflecting adaptation to a wide range of ecological conditions [6], [22]. This findings established the rational for exploring the genetic and chemical diversity of Sardinian populations of *H. italicum* without reference to the subspecies to which they belonged.

We report the application of the AFLP (amplified fragment length polymorphism) DNA fingerprinting platform in combination with essential oil analysis obtained by gas chromatography mass spectrometry (GC-MS), aimed at evaluating simultaneously the genetic and metabolite diversity of *H. italicum* populations sampled from disparate sites in Sardinia. The data should be informative for collection and conservation activities, as well as for the wider exploitation of the secondary metabolite content of the species.

## Materials and Methods

### *H. italicum* collection and sampling sites

Young stems of about 10 cm in length were collected from 294 *H. italicum* plants between March and July 2010 and stored at −20°C at the “Centro Interdipartimentale per la Conservazione e Valorizzazione della Biodiversità vegetale” (University of Sassari, IT). To avoid the collection of clonal material, only well spaced plants (at least 10 m apart from one to other) were sampled. When this was not possible the number of sampled individuals was reduced from 6 up to a minimum of 4 (Table 1). All plants were harvested at the full blooming stage.

**Table 1:** Sites chosen for the collection of Sardinian *H. italicum*.

Population	Zone	UTM E	UTM N	Locality	Alt*	n	Coverage
POP1	32S	457673	4350059	Monteponi	133	6	100mq
POP2	32S	451495	4349092	Fontanamare	0	4	100mq
POP3	32S	450396	4346517	Porto Paglia	0	6	100mq-1ha
POP4	32S	497324	4345282	Macchiareddu	17	6	100mq-1ha
POP5	32S	494244	4339075	Santa Lucia	75	6	100mq-1ha
POP6	32S	489732	4331633	Gutturumannu	263	6	100mq
POP7	32S	473958	4322928	Santadi	138	6	100mq
POP8	32S	465809	4313563	Porto Pino	3	6	100mq
POP9	32S	478085	4308660	Capo Malfatano	60	6	Isolated Plants
POP10	32T	472646	4527518	Lu Bagnu	20	6	100mq-1ha
POP11	32T	488084	4536341	Li Junchi	10	6	100mq
POP12	32T	497028	4544560	Costa Paradiso	11	6	100mq-1ha
POP13	32T	515899	4563956	Santa Teresa	46	6	100mq
POP14	32T	454916	4518708	Platamona	43	6	100mq-1ha
POP15	32T	447972	4520747	Porto Torres	20	6	100mq
POP16	32T	433215	4535543	Stintino	10	6	>1ha
POP17	32T	544563	4445847	Serra Ortenie (Oliena)	300	6	100mq
POP18	32T	535446	4457234	Oliena	670	6	Isolated Plants
POP19	32T	535382	4456459	Monte Maccione (Oliena)	730	6	Isolated Plants
POP20	32T	535132	4455787	Monte Maccione (Oliena)	800	6	Isolated Plants
POP21	32T	535556	4456138	Monte Maccione (Oliena)	900	6	Isolated Plants
POP22	32T	535629	4456012	Monte Maccione (Oliena)	1000	6	100mq-1ha
POP23	32T	543224	4445507	Passo genna silana	1010	6	>1ha
POP24	32T	543516	4459699	Valle di Lanaito	183	6	100mq-1ha
POP25	32T	524982	4462259	Nuoro	519	6	>1ha
POP26	32S	513670	4361793	Dolianova	180	6	100mq-1ha
POP27	32S	513823	4368155	Zona Ballao	322	6	100mq-1ha
POP28	32S	518667	4370897	Sant'Andre Frius	522	6	100mq-1ha
POP29	32S	528837	4376241	Crabonaxi	173	6	Isolated Plants
POP30	32S	533255	4379348	Riu Flumineddu	89	6	Isolated Plants
POP31	32S	535806	4382401	Sa cea manna	449	5	100mq-1ha
POP32	32S	542920	4380029	Monte Cardiga	672	6	Isolated Plants
POP33	32S	538414	4391076	Perdasdefogu	588	6	>1ha
POP34	32T	504243	4506257	Oschiri	210	6	>1ha
POP35	32T	517416	4515259	Berchidda	260	6	100mq-1ha
POP36	32T	531548	4505015	Alà dei Sardi	630	6	100mq-1ha
POP37	32T	529145	4518988	Monti	300	6	>1ha
POP38	32T	529079	4518535	Monti	300	6	>1ha
POP39	32T	529079	4518535	Monti	300	6	>1ha
POP40	32T	519132	4514376	Monti	230	6	100mq-1ha
POP41	32T	471684	4498457	Florinas	400	6	100mq-1ha
POP42	32T	465519	4497853	Florinas azienda	187	6	>1ha
POP43	32T	433696	4496201	Alghero	20	6	100mq-1ha
POP44	32T	528044	4428255	Gennargetu	1250	6	100mq-1ha
POP45	32T	466543	4441895	Santulussurgiu	900	6	n.d.
POP46	32T	469503	4440939	Camugheras	550	6	n.d.
POP47	32T	538022	4560202	Caprera	0	6	n.d.
POP48	32T	497691	4432185	Ortueri	580	6	100mq-1ha
POP49	32S	507488	4421096	Meana Sardo	600	5	100mq-1ha
POP50	32S	512299	4412642	Santa Sofia - Laconi	823	5	100mq-1ha

* The altitude is expressed as meter above sea level (m.a.s.l).

The geographical zone, geographic coordinated (UTM E and UTM N), the locality and the elevation above sea level of each site is given, along with the number of individuals sampled within each population (n), and the area of ground colonized by the population (mq).

**Figure 1:**
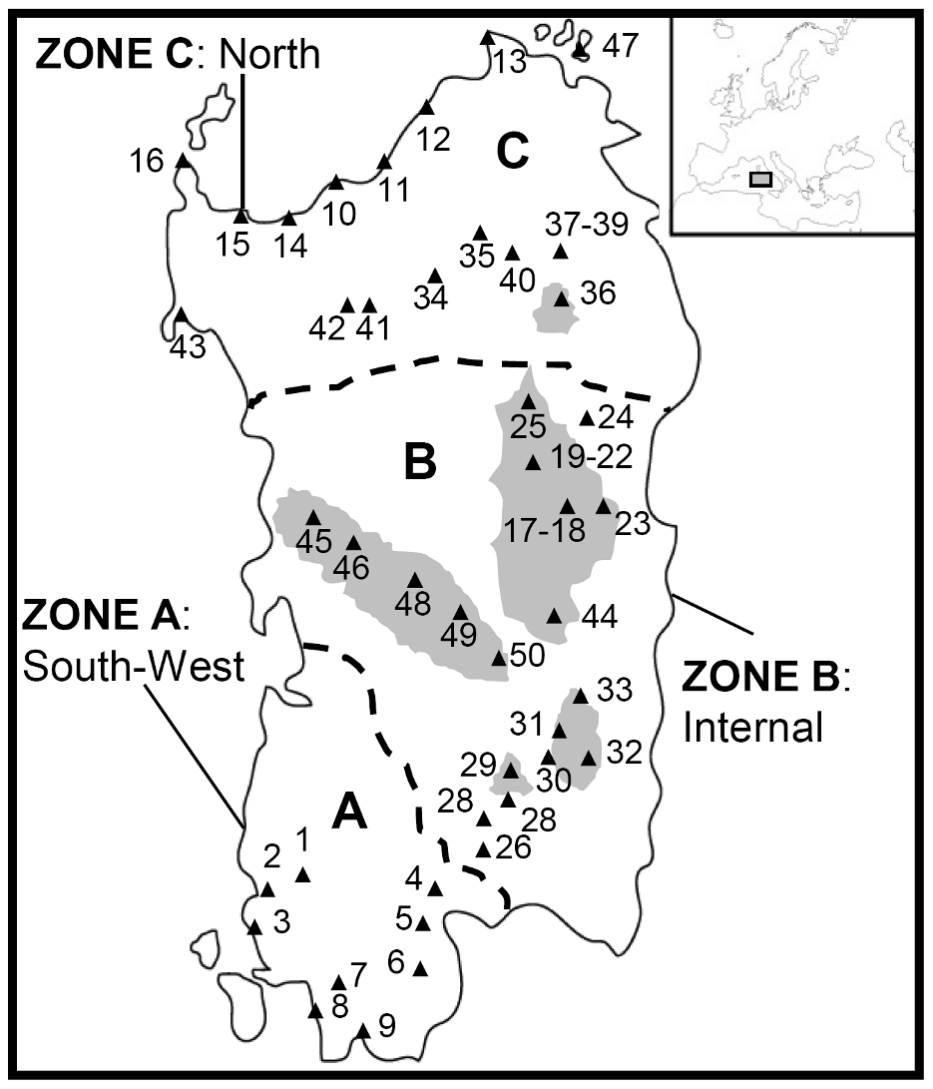
A map illustrating the location of the *H. italicum* collection sites. Sites shaded in gray indicate an elevation of >500 m a.s.l. The island was divided in three regions, namely zones A (southwestern), B (interior) and C (northern).

The location of the 50 collection sites (Figure 1) was determined by GPS, which along with other features of the sites, is recorded in Table 1. No endangered or protected species were involved and no specific permissions were required at any of the sites.

Meteorological data relevant for each site were provided by the Environmental Protection Agency of Sardinia (ARPAS), Hydrology, Meteorology and Climatology Department, derived from facilities located close to each site (Table S1 in File S1). Mean monthly parameters reflected historical (1997–2010) data. The habitat and soil type at each site are given in Table S2 in File S1.

### AFLP genotyping

Total genomic DNA was extracted using a DNeasy® Plant kit (QIAGEN, Hilden, Germany) following the manufacturer's protocol. AFLP analysis, based on the two restriction enzymes *Mse*I and *Eco*RI and the three primer combinations E-AAC/M-CAT, E-AGC/M-CTA and E-ACC/M-CAT, was carried out according to Vos et al. [23] with minor modifications. Thus, the genomic DNA (250 ng) was double digested with 5 U of EcoRI and MseI (New England Biolabs, Ipswich, MA, USA) in 10× Restriction/Ligation buffer (100 mM Tris base, 100 mM MgAc, 500 mM KAc, 50 mM DTT, 100 ng/µl BSA). Ten microliters of ligation mix (5 µM EcoRI adapters +1A, 50 µM MseI adapters +1C, 10 mM ATP, 1 U T4 ligase) was added to the restriction mix and incubated for 3 h at 37°C. A 5 µl aliquot of a 1∶9 dilution of this reaction represented the template for a 20 µl pre-amplification reaction containing 1.5 mM MgCl2, 2 µl 10× buffer, 10 mM dNTP, 2.75 µM each of the EcoRI and MseI primers and 1 U Taq polymerase. The resulting amplicon was diluted 1∶4 with water and a 5 µl aliquot was used as the template for a selective PCR, primed with one of the three EcoRI/MseI primer combinations E-AAC/M-CAT, E-AGC/M-CTA, E-ACC/M-CAT. All PCRs were performed using Platinum ® Taq DNA Polymerase High Fidelity. The amplicons were electrophoresed through 6% denaturing polyacrylamide gels, with the 100 bp DNA Ladder 100 (Invitrogen Life Technologies) included to allow for the estimation of fragment sizes. The fragments were visualized by silver staining, using a protocol adapted from Bassam et al. [24]. Fragments were scored on a presence (1)/absence (0) basis. Only strongly amplified fragments in the size range 80 bp-500 bp were considered.

### Population structure

The population structure was investigated using the Bayesian clustering model implemented in STRUCTURE 2.3.3 [25].

The settings were based on the recessive allele mode with an admixture model correlated to allele frequencies and no *a priori* information regarding population origin. The range in *K* considered was 1–15, and for each value of *K*, 20 replicate chains of 200,000 MCMC interactions were run with a burn length of 100,000. The most likely number of genetic clusters (*K*) was evaluated as suggested by Evanno et al. [26]. To incorporate geographical coordinates into the assignment analyses, the program TESS 2.3 [27] was run, employing an admixture model with 300,000 sweeps and a burn-in period of 100,000.

Again, the number of clusters studied ranged from 1 to 15, and 20 replicates were considered for each *K*.

### Population divergence

Population divergence was evaluated by analysis of molecular variance (AMOVA) as implemented in ARLEQUIN v3.5.1.2 [28]. Loci which were putatively either neutral or under selection loci were identified by BAYESCAN 1.0 software [29] (99% confidence interval, pilot run length 5,000). The loci identified were used for subsequent AMOVA and a Mantel test. A phylogeny was constructed based on UPGMA clustering [30], using TFPGA v1.3 software [31].

### Extraction, isolation and identification of essential oils

The essential oil samples were isolated from young stems by hydrodistillation in a Clevenger type apparatus for 1 h with 500 ml distilled water, following an established protocol [32]. Subsequent GC-MS analysis was carried out using a Hewlett Packard 5890 GC-MS system operating in EI mode at 70 eV and equipped with either (1) an HP-InnoWax capillary column (30 m×0.25 mm, film thickness 0.17 mm), over a temperature gradient of 4°C per minute, starting at 60°C for three minutes and ending at 210°C for 15 min; or (2) a HP-5 capillary column (30 m×0.25 mm, film thickness 0.25 mm) over a temperature gradient of 4°C per minute, starting at 60°C for three minutes and ending at 300°C for 15 min. The injection and transfer line temperatures were 220°C and 280°C, respectively. Helium was used as the carrier gas at a flow rate of 1 ml per minute and a split ratio of 1∶10. The identification of components was achieved by comparing the GC retention index (RI) on the apolar and polar columns with those of authentic samples of various essential oils and by matching the MS fragmentation patterns and retention index with stored Wiley 7 mass computer library, NIST (National Institute of Standards and Technology) or data in the literature [33], [34]. A hydrocarbon mixture of alkanes (C9-C22) was analyzed separately under the same chromatographic condition to calculate the RIs using a generalized equation [35]. The following standards were included: linalool (Purity ≥95%, Fluka), 1,8-cineole (99% purity, Aldrich), nerol (Purity ≥90%, Fluka), geraniol (Purity ≥96%, Fluka). C9-C22 alkane standards (purity 98–99%) were purchased from Aldrich.

### Statistical analysis

Correlations between metabolites, site altitude, site climate and the genetic coefficient of membership (*Q*) were calculated. A Pearson's χ^2^ test for 2×2 contingency tables was performed for the categorical variables soil type and habitat class [36] to test correlations with the clades identified by STRUCTURE [25]. Principal Component Analysis (PCA) was employed to reduce the complexity of the meteorological data. All variables were standardized for PCA analysis (Table S3 in File S1), and the analyses were carried out using JMP 7 software [36]. Climatic, geographic and genetic pairwise distance matrices were calculated for the purpose of the Mantel test. The climatic distance matrix was calculated by considering the first five principal components, based on the Euclidean method; geographical distance matrices between populations were computed from GPS coordinates, while genetic distance matrices were calculated in the form of *F_st_* [28]. The Mantel test and partial Mantel tests between geographic, climatic, and genetic distance matrices were performed using XLSTAT 2007 software [37].

## Results

### Population genetic structure and divergence

**Figure 2:**
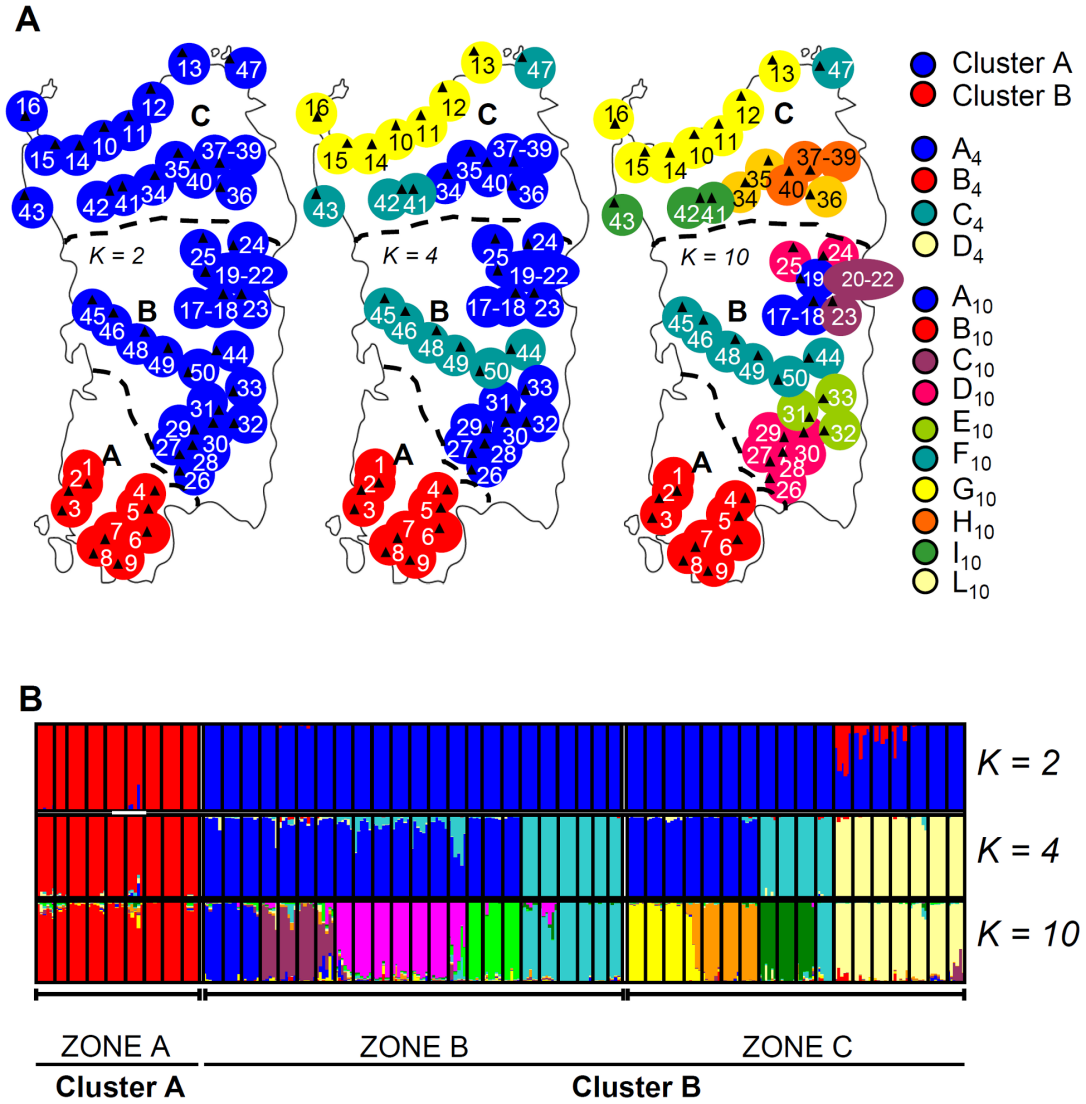
The genetic structure of the *H. italicum* population as predicted by various values of *K*. A) The geographical distribution of clades at *K* = 2 (clusters A and B), 4 (A_4_–D_4_) and 10 (A_10_–L_10_). The predominant clade at is colour coded at each level of *K*. B) *K* = 2 provides the optimum model. Each population is represented by a thin vertical line. White segments separate distinct geographical zones.

**Figure 3:**
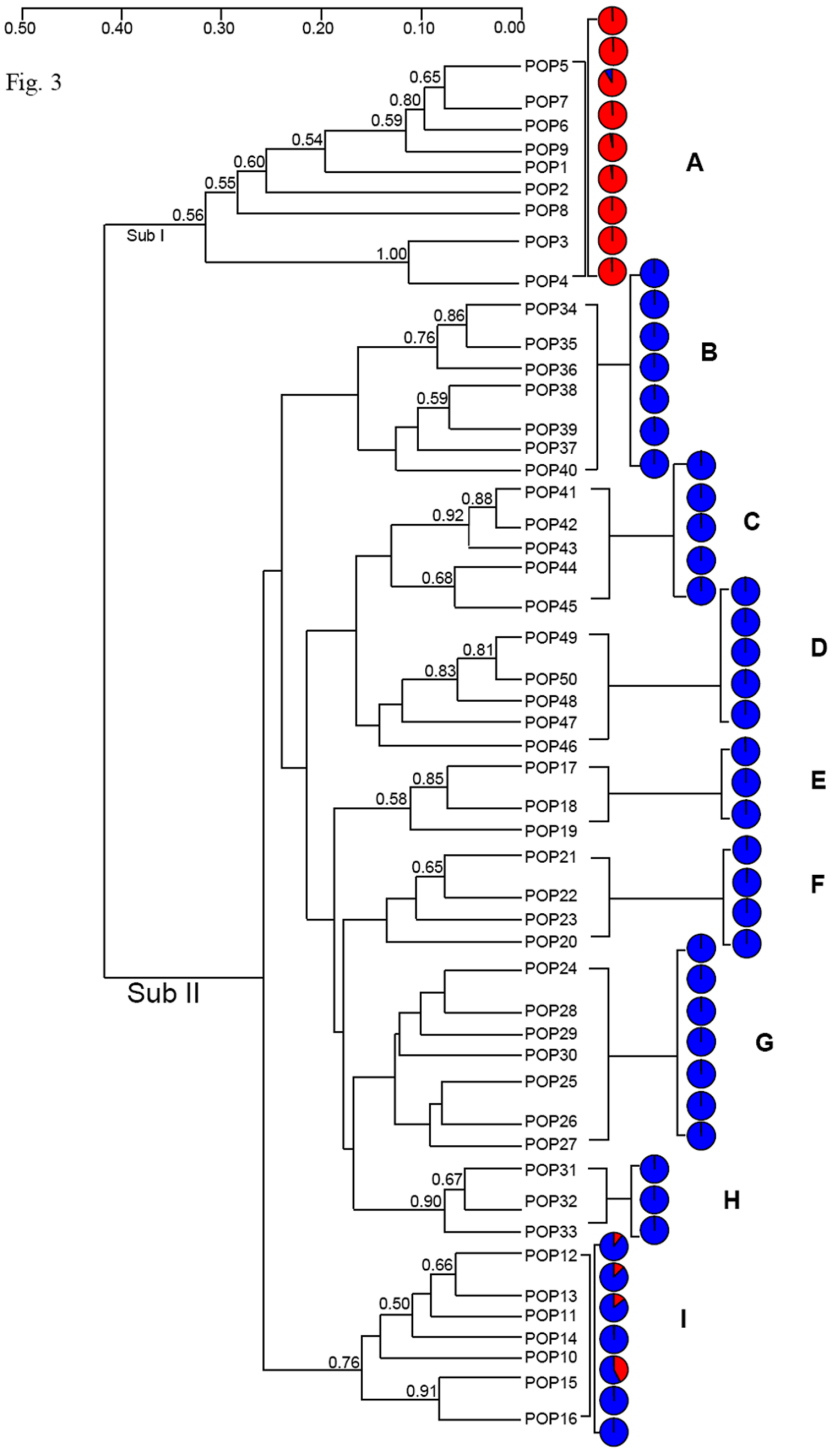
A UPGMA-based dendrogram illustrating the genetic relationships between the 50 *H. italicum* populations. Each individual pie chart indicates *Q*, the mean coefficient of membership of the population, at *K* = 2; The 1,000 replicate bootstrap support fractions are indicated for the higher nodes.

The genotypic data set comprised presence/absence scores for 125 AFLP fragments. STRUCTURE supported the presence of differentiation among the populations, and the *ΔK* method [26] identified two main clusters as the most likely structure (*ΔK* = 37.00, Figure S1 in File S1). The incorporation of spatial information using TESS software [27] similarly identified the populations to have a bipartite structure (Figure S1 in File S1). The clades defined by the two independent clustering methods were substantially congruent with one another (Figure S1 in File S1). Cluster A was populated by material originating from the southwestern region (zone A in Figure 1), while Cluster B was dominated by collection sites in the interior and northern regions (zones B and C, respectively). Zones B and C are separated from each other by the Marghine mountain chain, and zones A and B are interrupted by a region of intensive cultivation (Campidano). The coefficients of membership of individuals to the clades were quite high, except for samples from populations 10 through 16, which were collected from the northern coast (*Q* value of 0.88) (Figure S1 in File S1, Table S4 in File S1). As *K* was increased, the new sub-clades which formed all split off from Cluster B, with Cluster A remaining essentially intact (Figure 2 A, B). The initial populations to drop out of Cluster B were those collected in the northern interior and coastal regions, followed by materials originating from the interior region (Figs. 1, 2B). Inter-population relationships were graphically illustrated by a UPGMA-based dendrogram (Figure 3). This analysis showed that the two main groups, which were only 60% genetically similar to one another, corresponded fairly well to Clusters A and B (as identified by Bayesian clustering). Cluster B was divided in eight sub-clusters (labelled B through I in Figure 3).

The AMOVA showed that about one third of the molecular variance was attributable to the difference between Clusters A and B, a proportion which was not much altered when the higher order partitions (*K*>2) were tested (data not shown); a further one third of the molecular variance was explained by differences within each clade (Figure S2 in File S1). The pairwise distances between populations assigned to Cluster A members and members of Cluster B (Figure 2A, B) were greater than those between populations assigned to other clusters (Figure S2 in File S1). Several rather high pairwise distances obtained between members of Cluster B, as for instance between populations 34–36 and 31–33 (Figure 2A, B), which produced an *F_st_* of 0.63, compared to the *F_st_* of 0.54 between populations 31–33 and 17–19 (Figure S2 in File S1). The BAYESCAN 1.0 tool [29] identified 43 AFLP loci (34%) which exceeded the threshold log_10_ value of 2.0 (posterior odds probability >0.99) (Figure S3 in File S1). The molecular variance was then re-calculated considering either only the 43 loci putatively under selection or those which were neutral (Table 2). The variance based on the former was partitioned into 75.06% “between populations” and 24.94% “within populations”, while the apportioning of the variance based on neutral loci produced a picture similar to that obtaining for the marker set as a whole.

**Table 2:** AMOVA based on subsets of the AFLP data.

Alleles	Source of variation	d.f.*	Sum of squares	% Variation
Full set	Among population	49	3551.92	59.71
	Within population	244	1820.98	40.29
Loci under selection	Among population	49	1442.20	75.06
	Within population	244	384.20	24.94
Neutral loci	Among population	49	2109.73	51.77
	Within population	244	1436.78	48.23

* d.f. = degree of freedom.

“full set” includes all loci, “loci under selection” only loci deemed to be under putative selection, and “neutral loci” only loci deemed not to be under selection.

### Relationships between the collection site and genotype

**Figure 4:**
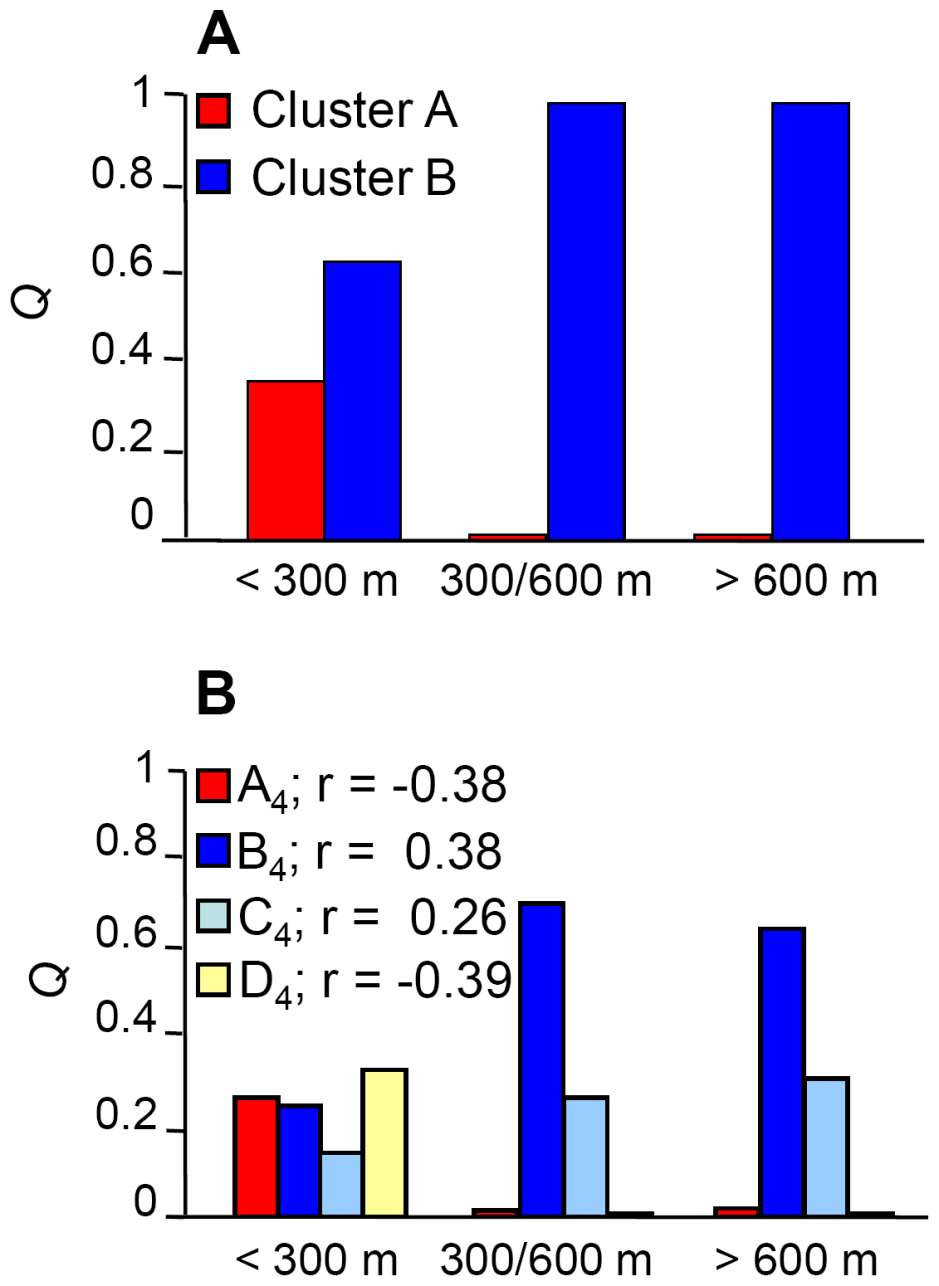
Average coefficient of membership (*Q*) for the lowland (<300 m a.s.l.), mid altitude (300–600 m a.s.l.) and highland (>600 m a.s.l.) populations. A) At *K* = 2, *Q* is significantly correlated with elevation (Cluster A, r = −0.42, P<0.0001, Cluster B, r = 0.42, P<0.0001). B) At *K = *4, members of Clusters A (identical to Cluster A at *K* = 2) and D predominate in the lowland sites, while members of Clusters B and C are found in the mid altitude and highland sites.

**Figure 5:**
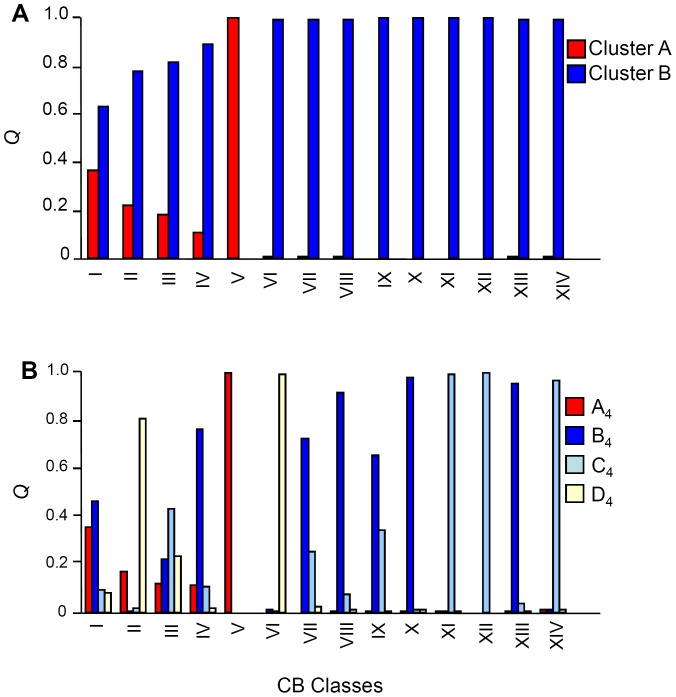
Average coefficient of membership (*Q*) with a set of 14 CORINE habitat classes. A) At *K* = 2, Cluster A members occur in habitats I through V, but are absent in VI through XIV. Cluster B members are found in all except one of the habitats (V). B) At *K* = 4, Cluster A members behave as for *K = *2, but Cluster B is split into three distinct groups.

**Figure 6:**
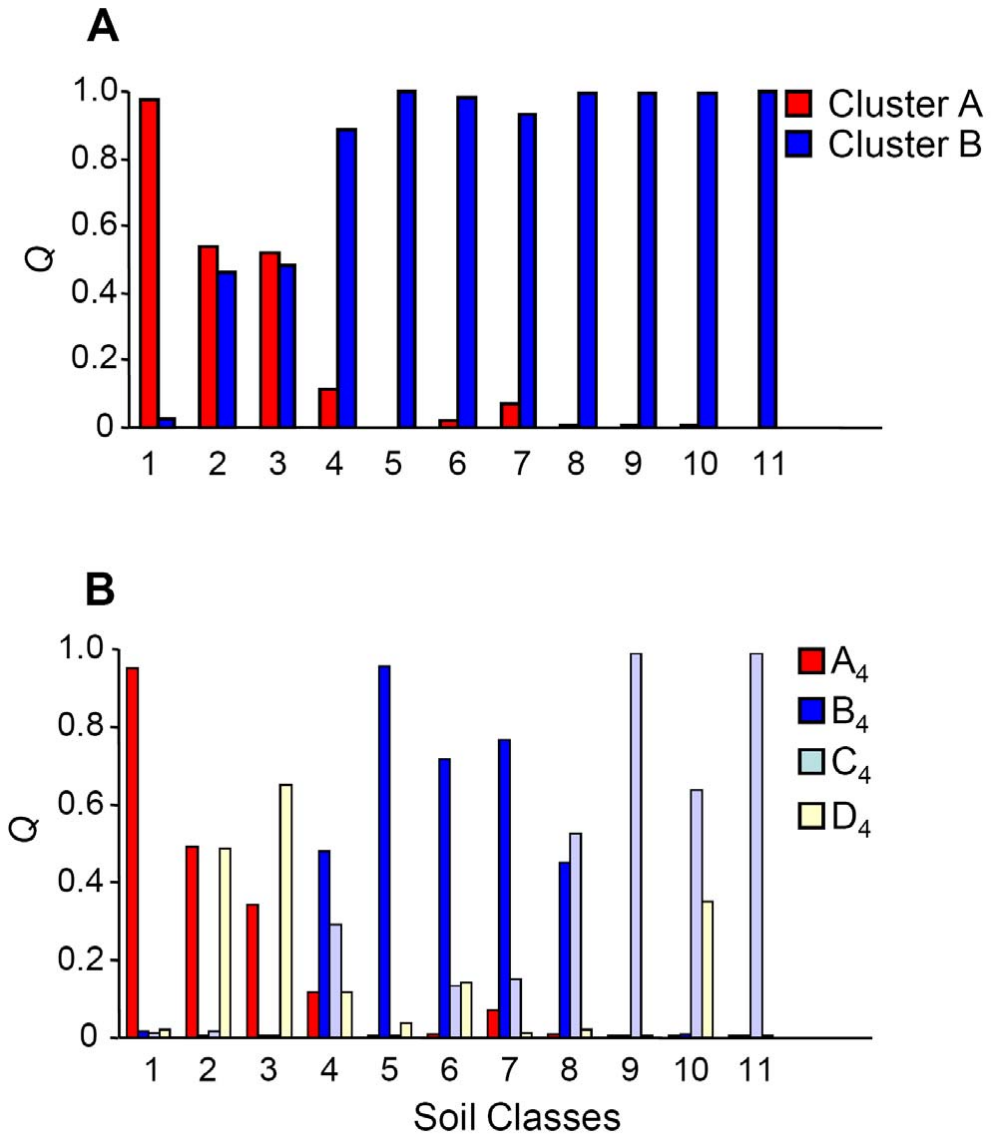
Average coefficient of membership (*Q*) with a set of 11 soil classes. A) *K = *2, B) *K = *4.

The congruence between the AFLP-based clustering and accession origin prompted a consideration of the relationship between genetic diversity and aspects of the collection sites' physical environment. The sites were first grouped on the basis of their elevation above sea level into “lowland” (<300 m), “mid altitude” (300–600 m) and “highland” (>600 m) and the AMOVA was then repeated. Partitioning of the genetic variance showed that 4.90% resided between elevation classes, 55.48% between populations within an elevation class and 39.54% within populations of same altitude class (Table 3). When the germplasm was re-organized to fit a *K* = 4 model (Figure 2), Cluster A_4_ and D_4_ members all fell into the lowland sites (Figure 4A, B), while Cluster B_4_ and C_4_ members dominated the mid and higher altitude class (Figure 4B). The CORINE-based classification of habitat type defined 14 habitats (Table S2 in File S1) [38]. At first glance, there was little evidence of any relationship between AFLP-based clade and habitat (Figure 5A, B), but it was noticeable that Cluster A members dominated the salt pioneer swards habitat (Figure 5A). The resulting hierarchical AMOVA showed that 3.26% of the molecular variance could be explained by habitat type (Table 3). A χ^2^ test indicated a non-random distribution between AFLP clade and habitat (P<0.0001), a result which was strengthened when the rarer habitats were excluded (Table S5 in File S1). The next physical aspect of the collection sites to be tested was soil type (Table S2 in File S1) [39]. The AMOVA indicated that 9.38% of molecular variance could be explained by this factor, and the subsequent χ^2^ test again showed a non-random association between soil type and AFLP clade (Table S5 in File S1). The distribution of the populations with respect to soil type is shown in Figure 6. Note that the sole colonizers of gleyic solonchak soils were Cluster A members, some of which were also sampled from gleyic arenosol and haplyic nitosol soils. Group C_4_, a population derived from cluster B (see Figure 2A, B), dominated rocky outcrops, but was also represented on some other soil types, while the haplic soils were preferentially populated by individuals belonging to group D_4_ (derived from cluster B, *K* = 2) (Figure 6B).

**Table 3:** Hierarchical AMOVA to determine the influence on the genetic structure of geographic location of collection site, its elevation above sea level, its soil type and its class of habitat.

Population	Source of variation	d.f.	Variance component	% Variation	*F_st_*
Overall	Among population	49	11.06	59.71	0.60
	Within population	244	7.46	40.29	
Geographic zone (A, B, C)	Among zones	2	3.75	18.88	0.62
	Among populations within zone	47	8.66	43.57	
	Within populations of the same geographic zone	244	7.46	37.54	
Altitude Classes (AC)	Among AC	2	0.94	4.99	0.60
	Among populations within AC	47	10.47	55.48	
	Within populations of the same AC	244	7.46	39.54	
Habitat classes (CB)	Among CB	13	0.61	3.26	0.59
	Among populations within CB	36	10.54	56.64	
	Within populations of the same CB	244	7.46	40.10	
Soil Classes (SC)	Among SC	10	1.72	9.38	0.59
	Among populations within SC	37	9.12	49.72	
	Within populations of the same SC	234	7.50	40.90	

### Correlation between genetic distance and local climate

Relevant meteorological data for the various collection sites are given in Table S1 in File S1 and the five principal components (PCs) identified by the PCA in Table S3 in File S1. The first PC (PC1) explained about half of the overall meteorological variance and was positively correlated with temperature and negatively with rainfall, while PC2 (19% of the variance) was dominated by the mean maximum summer temperature. The remaining three PCs each explained between 5% and 12% of the variance, and the first five components together more than 90% of the variance. The pairwise climatic distances between sites were expressed as a Euclidean distance based on PC1 through PC5 and are here referred to as ecological distances. According to a Mantel test, the pairwise genetic distances were positively correlated with both climate and geographic separation (Table 4). The latter two variables were also highly correlated with one another. The correlation between genetic and climatic distance decreased by one third when controlling for geography. A similar result obtained for the correlation between genetic distance and geographical separation when the effect of climate was factored out. When the correlations were re-calculated to reflect the set of AFLP loci which were either neutral or putatively under selection (Table 4), the one between genetic distance and ecological distance was reduced by about one third when the neutral markers were considered, while it rose by a third for the set of loci which were putatively under selection. The suggestion is that the distribution of genetic variation has been shaped by natural selection, but in spite of being correlated with ecology, geographical location on its own has still been an important factor. The correlation between genetic distance and geography was statistically significant even after controlling for the effect of ecology for both the neutral markers and those putatively under selection.

**Table 4:** Mantel test to evaluate the correlation between the sites' geographic locations, ecologies and genetic distance.

Allele	Matrices	Pearson	Pvalue
All alleles	Geographic vs Ecological	0.24	**<0.0001**
	Genetic vs Geographic	0.18	**<0.0001**
	Genetic vs Ecological	0.22	**<0.0001**
	(Genetic vs Geographic)-Ecological	0.14	**<0.0001**
	(Genetic vs Ecological)-Geographic	0.18	**<0.0001**
Neutral alleles	Geographic vs Ecological	0.24	**<0.0001**
	Genetic vs Geographic	0.18	**<0.0001**
	Genetic vs Ecological	0.15	**<0.0001**
	(Genetic vs Geographic)-Ecological	0.15	**<0.0001**
	(Genetic vs Ecological)-Geographic	0.11	**<0.0001**
Alleles under selection	Geographic vs Ecological	0.24	**<0.0001**
	Genetic vs Geographic	0.27	**<0.0001**
	Genetic vs Ecological	0.29	**<0.0001**
	(Genetic vs Geographic)-Ecological	0.22	**<0.0001**
	(Genetic vs Ecological)-Geographic	0.24	**<0.0001**

The same test was repeated on the subsets of marker data referred to in Table 3.

### Essential oil composition

**Figure 7:**
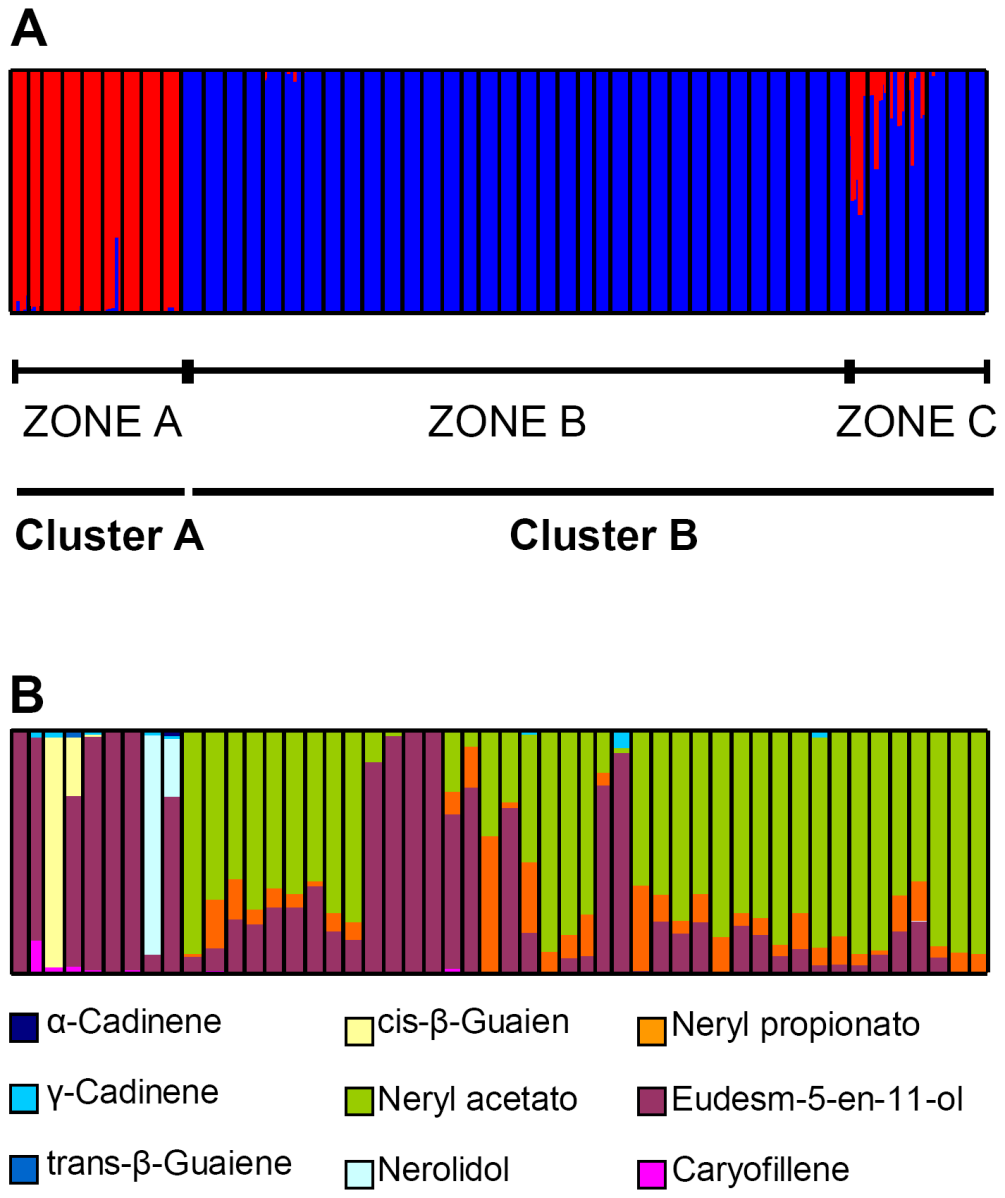
Pairwise correlations between average coefficient of membership (*Q*) in Cluster A and essential oil components. A) The genetic structure identified at *K* = 2, compared to the compounds significantly correlated to genetic structure. B) The geographically and genetically derived clusters are largely congruent with the essential oil component chemical profiles.

The GC-MS analysis highlighted 35 distinct compounds, comprising five monoterpenes, ten oxygenated monoterpenes, 15 sesquiterpenes and five oxygenated sesquiterpenes (Tables S6–S9 in File S1). A representative chromatogram is given in Figure S4 in File S1. The concentration of some of these compounds varied among the accessions. For instance, nerol and its derivatives were absent from populations 1–9, but were present in significant amounts in population 16. To derive the relationship between essential oil profile and AFLP clade, the compounds were initially grouped into the above four terpene classes. A contingency analysis showed that only the oxygenated monoterpenes were correlated with the average coefficient of membership at *K* = 2 (Table 5). When the concentration of each of the 35 compounds was compared separately, nine proved to be significantly correlated to AFLP clade at *K* = 2 (Table 6). The relationship between the concentration of these nine compounds and the genetic structure of the material at *K* = 2 is shown in Figure 7A and B. Two predominant chemotypes could be recognized, based on the presence/absence of eudesm-5-en-11-ol and neryl acetate. The populations collected from zone A (Cluster A members) produced significant levels of eudesm-5-en-11-ol and almost negligible quantities of the other compounds (Chemotype 1), while samples collected in zones B and C (populations 9 through 50, Cluster B members) contained substantial quantities of neryl acetate, but relatively little eudesm-5-en-11-ol (Chemotype 2) (Figure 7 B).

**Table 5:** Correlation analysis between the AFLP-based clades (Clusters A and B) and the essential oil profile.

Chemical Group	χ2	Prob
Monoterpens	0.076	0.078
Oxygenate Monoterpens	16.463	<0.001
Sesquiterpens	0.027	0.869
Oxygenated Sesquiterpens	0.457	0.499

The latter were classified into four major chemical groups.

**Table 6:** Pairwise correlation between AFLP-based clades (Clusters A and B) and essential oil.

Variable	Correlation (r)	P value
Caryofillene	0.53	<.0001
Eudesm-5-en-11-ol	0.37	0.0102
Neril propionato	−0.50	0.0002
Nerolidol	0.36	0.0106
Neril acetato	−0.55	<.0001
cis-β-Guaiene	0.36	0.0099
trans-β-Guaiene	0.30	0.0322
α-Cadinene	0.50	0.0002
γ-Terpinene	0.31	0.0308

Only compounds significantly correlated to the genetic structure are included.

## Discussion

AFLP profiling of the Sardinian populations of *H. italicum* strongly supported the existence of two major clades. The coefficient of membership of individuals to either cluster was, in general, rather high. For the most part, the AFLP-based clades were congruent with the collection site of the populations. The only exception to this scenario was the admixture noticed in the northern coast populations. The suggestion is therefore that gene flow between the various populations is rather limited. *H. italicum* is an allogamous species which relies on insects for pollination. A reduced mobility of pollinators may have been responsible for the limited gene flow observed between the populations. Note particularly that the membership of Cluster A was hardly affected by increasing the *K* value, although this was not the case for Cluster B. The indication is thus that a bipartite genetic structure is an intrinsic feature of Sardinian *H. italicum* populations. This conclusion needs to be viewed in the light of the proposal that the two *H. italicum* ssp. are part of the same gene pool, and that local populations have become differentiated by natural selection [5]. The congruence between genetic structure and the physical environment is suggestive of the diversity being shaped by adaptive selection. Cluster A was largely restricted to lowland sites, while Cluster B members were concentrated in the mid altitude and highland sites. In contrast, Galbany-Casals et al. [22], [40] have proposed that the species' genetic structure in the western and central Mediterranean Basin takes the form of a continuous gradient in allele frequency rather than exhibiting any sharp division to form distinct populations; they describe a high correlation between geographic and genetic inter-population distances, interpreted as reflecting a typical pattern of isolation by distance. The apparent discrepancy with the present findings may, at least in part, be accounted for by the choice of sampling method. Since the Galbany-Casals et al. [5] experiments were designed to address whether ssp. *italicum* and ssp. *microphyllum* had evolved independent gene pools, their focus was on morphological intermediates. In contrast, the present approach based its sampling on as wide as possible a range of ecological conditions. A risk of this strategy is the introduction of an unintended bias against sites in transitional zones. Working at a single population level led to the recognition of a positive correlation between genetic distance and geographical separation, implying the action of dispersive forces and limited gene flow. Nevertheless, the full analysis indicated that the genetic distance between populations was better explained by local ecology rather than by geographical separation. The sampling of the marker set to include loci presumed to be under selection indicated that these too tended to be associated with the climatic variables, underlining the contribution of adaptive selection.

The composition of the essential oils was rather uniform within, but differed markedly between the populations. The profile of Italian populations of *H. italicum* is reportedly quite variable [21], but as yet there has been no attempt to correlate it with either genetic diversity or ecological/environmental variables. Three major chemotypes have been reported in the literature [4], [19], but much of the variability observed was ascribed to geographical origin and flowering time. With respect to the Sardinian populations, only limited information of this type has until now been documented [41]. The present data show for the first time, that the presence/absence of specific oxygenated monoterpenes was consistent with the genetic structure of the populations, as defined by AFLP fingerprinting. As already described elsewhere for Corsican and Sardinian *H. italicum* populations [42], the predominant essential oil components were neryl acetate, nerol, neryl propionate and eudesm-5-en-11-ol. The presence/absence of neryl acetate and eudesm-5-en-11-ol were well correlated with the *K* = 2 genetic structure of the populations, although some of the other compounds also showed some correlation.

The data strongly support the proposition that the two AFLP-based clades identified are associated with both ecological factors (such as altitude, soil type and habitat) and specific chemotypes (Figure 7). The association between genetic structure and the physical properties of the sampling site prevented any analysis of the relative importance of location and genotype on the determination of the essential oil profile. The association between chemotype and collection location will need to be considered when elaborating a strategy for any future collection exercise aimed at identifying valuable sources of essential oil.

## Supporting Information

File S1 Supporting figures and tables. Figure S1, Admixture proportions at *K = 2* of 294 *H. italicum* individuals sampled from 50 populations. A Bayesian clustering analysis was performed using STRUCTURE and TESS software. The highest likelihood run was identified based on minimum values for Δ*K* [26] and DIC [27], respectively. Figure S2, Pairwise *F_st_* between the AFLP-based clades identified at A) *K* = 4 and B) *K* = 10. At *K = 4*, clades A and B were the most divergent, but at *K = 10*, they were replaced by clades G and I. Figure S3, The identification of AFLP loci putatively under selection loci using Bayescan 1.0 software [29]. Each point corresponds to a single AFLP fragment. *F_st_* is plotted against the log_10_ of posterior odds. The vertical dashed line indicates the chosen threshold value of 2.0. Figure S4, Representative chromatogram of the Essential oil of *H. italicum*. The main peak in the chromatogram represents neryl acetate. Table S1, Location of the meteorological stations, in terms of their latitude, longitude, elevation and distance from the sea. Table S2, Soil and habitat description. For each population the collection site was described for habitat, CORINE Biotope code (CB) and soil. Habitats and soils description is shown the corresponding columns. The habitats as well as the soils were divided in groups with similar characteristics. Fourteen CB groups (I to XIV) and ten soil types (1–10) were identified; missing data are indicated as n.d. Table S3, The first five PCs explaining >90% of the variation in climate, reduced from a set of 48 climate parameters. Significant correlations between each PC and individual parameters are indicated in bold. Air temperature and rainfall amounts are annual averages. Table S4, Coefficient of membership (*Q*) with the major clades of individual accessions of *H. italicum*. A) *K = *2, B) *K* = 4, C) *K = *10. Table S5, Contingency analysis for habitat and soil type with respect to AFLP-based clades at *K* = 2 and *K* = 4. The analysis was repeated with a restricted set of habitat and soil types. Table S6, The monoterpene fraction of *H. italicum* essential oil. Table S7, The oxygenated monoterpene fraction of *H. italicum* essential oil. Table S8, The sesquiterpene fraction of *H. italicum* essential oil. Table S9, The oxygenated sesquiterpene fraction of *H. italicum* essential oil.
(RAR)
